# Association of the components of the metabolic syndrome with non- alcoholic fatty liver disease among normal-weight, overweight and obese children and adolescents

**DOI:** 10.1186/1758-5996-1-29

**Published:** 2009-12-22

**Authors:** Roya Kelishadi, Stephen R Cook, Atoosa Adibi, Zahra Faghihimani, Shohreh Ghatrehsamani, Abolfazl Beihaghi, Hamidreza Salehi, Noushin Khavarian, Parinaz Poursafa

**Affiliations:** 1 Associate Professor of Pediatrics, Pediatric Preventive Cardiology Department, Isfahan Cardiovascular Research Center, Isfahan University of Medical Sciences, Isfahan, Iran; 2 Assistant Professor of Pediatrics, Department of Pediatrics, University of Rochester Medical Center, Rochester, NY, USA; 3 Associate Professor of Radiology, Radiology Department, Isfahan University of Medical Sciences, Isfahan, Iran; 4 Research Assistant, Pediatric Preventive Cardiology Department, Isfahan Cardiovascular Research Center, Isfahan University of Medical Sciences, Isfahan, Iran; 5 Assistant of Radiology, Radiology Department, Isfahan University of Medical Sciences, Isfahan, Iran

## Abstract

**Objectives:**

This study aimed to determine the prevalence of the metabolic syndrome, abnormalities of liver enzymes and sonographic fatty liver, as well as the inter-related associations in normal weight, overweight and obese children and adolescents.

**Methods:**

This cross-sectional study was conducted among a sample of 1107 students (56.1% girls), aged 6-18 years in Isfahan, Iran. In addition to physical examination, fasting blood glucose, serum lipid profile and liver enzymes were determined. Liver sonography was performed among 931 participants. These variables were compared among participants with different body mass index (BMI) categories.

**Results:**

From lower to higher BMI category, alanine aminotransferase (ALT), total cholesterol, LDL-cholesterol, triglycerides and systolic blood pressure increased, and HDL-cholesterol decreased significantly. Elevated ALT, aspartate aminotransferase (AST) and alkaline phosphatase (ALP) were documented in respectively 4.1%, 6.6% and 9.8% of normal weight group. The corresponding figure was 9.5%, 9.8% and 9.1% in overweight group, and 16.9%, 14.9% and 10.8% in obese group, respectively. In all BMI categories, ALT increased significantly by increasing the number of the components of the metabolic syndrome. Odds ratio for elevated liver enzymes and sonographic fatty liver increased significantly with higher number of the components of the metabolic syndrome and higher BMI categories before and after adjustment for age.

**Conclusions:**

Because of the interrelationship of biochemical and sonographic indexes of fatty liver with the components of the metabolic syndrome, and with increase in their number, it is suggested to determine the clinical impact of such association in future longitudinal studies.

## Introduction

Overweight and obesity in children have become a worldwide problem, no more limited to high-income countries; among developing countries, the Middle East has one the highest rates of childhood obesity [1]. In Iran, the national prevalence of overweight and obesity among 6-18-year-old students is reported to be 13.4%, with large variations between different provinces [2], and as high as 28.9% in students aged 11-18 years in the capital city [3].

Metabolic syndrome, which is a constellation of metabolic abnormalities including insulin resistance, hyperinsulinemia, glucose intolerance, dyslipidemia, and hypertension, is common in overweight youths, affecting around 30% of overweight children and adolescents in the United States [4]. It probably plays an important role in developing type 2 diabetes and atherosclerotic cardiovascular diseases [5]. Nonalcoholic fatty liver disease (NAFLD) is another consequence of obesity and is related metabolic disorders [6]. Since obesity and diabetes are the two most common risk factors for NAFLD development, they can, at least partially, induce peripheral insulin resistance. However, an independent association of NAFLD with insulin resistance, as suggested by epidemiologic studies [7], has been confirmed through the use of the clamp technique. NAFLD includes different ranges of liver pathologies and outcomes from simple steatosis to nonalcoholic steatohepatitis (NASH) [6]. The latter could be more severe in children from special ethnic groups, such as Hispanics and Asians [8]. NAFLD usually develops in children who are obese [9]. While abnormalities of liver enzymes in pediatric NAFLD are moderate [8], a correlation between degree of obesity and severity of hepatic steatosis at ultrasonography has been reported [9,10]. Among adults, NAFLD markers such as alanine aminotransferase (ALT) might predict metabolic syndrome, independently. Such experience is very limited in the pediatric age group. A study in children and adolescents found a strong association of the metabolic syndrome with elevated ALT levels, and this association existed in a graded fashion across the number of metabolic components [11]. Thus, early diagnosis of overweight in children is a valuable goal in order to limit obesity development as soon as possible [8]. Although Asian children are suggested to have an ethnic predisposition to increased waist circumference [12] and metabolic disorders notably dyslipidemia even in those with normal weight (13, 14), data about the association of NAFLD and metabolic syndrome in children are scarce not only in Iran, but in Asia. The present survey aimed to collect relevant data on the prevalence of metabolic syndrome, abnormalities of liver enzymes and sonographic fatty liver, as well as the inter-related associations in a representative sample of overweight/obese children and adolescents. To the best of our knowledge, this is the first study of its kind not only in Iran, but also in the Eastern Mediterranean region, and the findings might be used for future ethnic comparisons.

## Methods

This cross-sectional population-based study was conducted in 2006-2007 in Isfahan, the second large city in Iran. It met the ethical guidelines of the Declaration of Helsinki and was approved by the Ethics Committee of Isfahan Cardiovascular Research Center, Isfahan University of Medical Sciences (ICRC-IUMS) (NIH Code: FWA 0000t8578).

### Study population

The target population was school students in Isfahan aged 6 to 18 years. After necessary arrangements with authorities of the Provincial Education and Training office, the project team selected the students by multistage-random cluster sampling from elementary, intermediate and high schools in different areas of Isfahan. Initially, the project team randomly selected sectors based on the data of the Provincial Education and Training office. Then, schools were stratified according to the location within the sectors, taking into account the proportion of different types of schools (public or private) to avoid socioeconomic bias. In each randomly selected school, a proportional two-stage cluster sample of students was selected. Then, schools were the primary units (clusters) and students within the schools were the secondary units. Students were coded and were randomly selected using random number tables.

Those with mental retardation, any chronic medical problem, long-term medication use for any health disorder, signs compatible with genetic syndromes as abnormal face, history of any kind of infectious or non-infectious hepatic disorder were not included to the study. As alcohol use is forbidden in our country, none of the individuals had used alcoholic beverages.

In the first step, weight and height of 7554 randomly selected students were measured in their schools [15], and after calculation of their body mass index (BMI.) as weight (Kg) divided by height squared (m^2^), they were categorized into three groups of normal weight, overweight (BMI of 85 to 94^th ^percentile) and obese (BMI equal or greater than 95^th ^percentile) based on the BMI charts of the US centers for disease control and prevention [16]. Then a random sample was selected from each BMI group, and students were included in the study after full explanation of the study protocol and obtaining written informed consents from parents and oral assents from students. The whole study was provided free of charge.

### Anthropometric measurements

Selected students were invited to the Pediatric Preventive Cardiology Department, (ICRC-IUMS). In order to confirm the BMI category, height and weight were twice measured to the nearest 0.2 cm and 0.2 kg, respectively, by using calibrated scale and stadiometer (Seca, Japan) with subjects being barefoot and lightly dressed, and the averages were recorded. The anthropometric measures were in agreement with those obtained in schools. All measurements were made by the same trained team of general physicians and nurses under the supervision of the same pediatrician. After filling out a questionnaire on demographic characteristics, the waist circumference (WC) was measured with a non-elastic tape at a point midway between the lower border of the rib cage and the iliac crest at the end of normal expiration. Hip circumference (HiC) was measured at the widest part of the hip at the level of the greater trochanter to the nearest 0.5 cm.

### Blood pressure measurements and biochemical analysis

Blood pressure (BP) was measured using mercury sphygmomanometers (Richter, Germany), after 5-minutes of rest in the sitting position. At this time, the procedure was explained to the students. They were seated with the heart, cuff, and zero-indicator on the manometer at the observer's eye level. All readings were taken in the right arm. Appropriate size cuffs were used with cuff-width 40% of mid-arm circumference, and cuff bladders covering 80-100% of the arm circumference and approximately two thirds of the length of the upper arm without overlapping. The first measured BP was not used in the analysis of this study. The reading at the first and the fifth Korotkoff phase was taken as systolic and diastolic BP (SBP and DBP), respectively. The average of the two time measurements was recorded and included in the analysis.

Then, while one parent accompanied his/her child, fasting venous blood sample was taken from the antecubital vein between 8:00 to 9:30 am. Serum fasting blood glucose (FBG), alanine aminotranferease (ALT), aspartate aminotransferase (AST), alkaline phosphatase (ALP), triglycerides (TG), total cholesterol (TC), low and high density lipoprotein cholesterol (LDL-C, HDL-C) were measured on fresh samples by standard kits (Pars Azmoun, Tehran, Iran) by using auto-analyzer (Hitachi, Japan). All Laboratory measurements were performed in ICRC central laboratory with adherence to external national and international quality control.

### Sonographic evaluation

Participants were referred to the Radiology ward of Nour hospital (formerely named Khorshid), affiliated to IUMS. We have previously reported details of sonographic examination [17], and here we report it in brief. Children and adolescents underwent ultrasound examination by two radiologists by using an ultrasound multi-frequency curvilinear 3.5-5 MHZ probe by Siemens Company (Sonoline G50 series, model number 7474922).

### Definition of elevated liver enzymes, metabolic syndrome and sonographic fatty liver

According to the study design, we had included nearly similar proportions of normal-weight, overweight and obese participants, therefore the percentiles of liver function tests were determined in a sub-sample of participants with a BMI distribution similar to the proportions documented in the whole 7554 students studied with a prevalence of 9.34% of overweight and 5.33% of obesity as we described before [15]. Thus, we randomly selected a similar proportion of participants from the 1107 individuals who underwent blood sampling for liver function tests by including 514 children and adolescents consisting of all normal-weight (n = 438), 48 overweight and 28 obese participants. ALT, AST and ALP were considered elevated if their levels were at or above the 90^th ^percentile value calculated from this sample population.

We used the definition of the International Diabetes Federation for the metabolic syndrome in children and adolescents [18]. WC above the age- and gender-specific 90^th ^percentile of Iranian children and adolescents was considered as abdominal obesity [19]. As described before [16], sonographic fatty liver was defined as increased echogenicity of the liver parenchyma to the extent that it was reported by ultrasound and disturbed the visibility of the portal vein and liver artery [20].

### Statistical analysis

Data were analysed by SPSS version 15.0 (SPSS Inc., Chicago, USA). Descriptive data were expressed as mean values ± standard deviations (SD) for continuous variables. Differences between genders were assessed by Student t test. The mean values of variables studied were compared between normal weight, overweight and obese groups by analysis of variance (ANOVA) and *post hoc *analysis. ANOVA and Kruskal Wallis tests were used for comparison of mean values of ALT, AST and ALP by the number of components of the metabolic syndrome in children and adolescents in the three groups of body mass index.

Correlation of waist circumference and cardio-metabolic risk factors with liver enzymes was determined according to sex and body mass index by using Pearson correlation analysis.

Logistic regression analysis was performed to determine the risks of elevated liver enzymes and sonographic fatty liver according to the number of components of the metabolic syndrome. The odds ratios (OR) and 95% confidence intervals (CI) for elevated liver enzyme levels according to the number of components of the metabolic syndrome and according to BMI categories are presented before and after age-adjustment. P < 0.05 was considered statistically significant.

## Results

The study participants comprised 1107 students including 621(56.1%) girls and 486 (43.9%) boys with a mean (SD) age of 12.57 (3.3) years. Of them, 331(29.9%) were in the 6-9.9-year age group, 426(38.5%) and 350(31.6%) were in the 10-13.9-year and the 14-18-year -age groups, respectively. Overall 438(39.5%) had normal weight, 377(34%) were overweight and 295(26.6%) were obese. Of the total population studied, 931 participants including 413 (44.4%) boys and 518(55.6%) underwent sonography.

The mean value of the variables studied was not significantly different in terms of gender; the only significant difference was documented for liver enzymes: the mean ALT, AST and ALP were significantly higher in girls than in boys (19.50 ± 10.4 vs. 17.98 ± 9.74, 24.04 ± 10.5 vs. 22.60 ± 10.15 and 525.43 ± 248.7 vs. 477.49 ± 269.56 U/L, respectively, p < 0.05).

As presented in Table 1, from lower to higher BMI category, anthropometric measures, ALT, total cholesterol, LDL-C, TG and SBP increased, and HDL-C decreased significantly. AST was not significantly different between normal weight and overweight group, but was significantly higher in obese group than in others; DBP was not significantly different between overweight and obese groups, but was lower in the normal weight group than in others. FPG and ALP were not significantly different between groups. Correlations of waist circumference and cardio-metabolic risk factors with liver enzymes are presented in Table 2, and shows significant correlations in different BMI categories. Elevated ALT, AST and ALP levels were documented in respectively 4.1%, 6.6% and 9.8% of normal weight group. The corresponding figure was 9.5%, 9.8% and 9.1% in overweight group, and 16.9%, 14.9% and 10.8% in obese group, respectively.

**Table 1:** Characteristics* of the study population according to the body mass index category

Variables	Total	Normal weight	Overweight	Obese	P
**Age **(years)	12.57 ± 3.3	12.08 ± 3.5	13.40 ± 3	12.23 ± 3.1	0.1
**Weight **(Kg)	51.37 ± 19.2	37.46 ± 13.1	56.07 ± 14.3	66 ± 18.7	<.0001
**Height **(cm)	151.11 ± 14.7	147.28 ± 16.4	154.19 ± 12.7	152.86 ± 13.3	<.0001
**Body mass index **(Kg/m^2^)	21.75 ± 5.5	16.65 ± 2.8	23.12 ± 2.9	27.59 ± 3.8	<.0001
**Waist circumference **(cm)	79.23 ± 14.4	66.71 ± 8.8	82.74 ± 8.8	93.33 ± 10.9	<.0001
**Hip circumference **(cm)	91.22 ± 14.6	79.84 ± 11.8	95.70 ± 10	102.39 ± 11.3	<.0001
**Alanine aminotransferase **(U/L)	19.50 ± 10.4	15.87 ± 7.8	19.62 ± 10.6	24.73 ± 11.3	<.0001
**Aspartate transaminase **(U/L)	24.04 ± 10.5	23.46 ± 9.2	23.33 ± 10.6	25.80 ± 11.8	0.003
**Alkaline phosphatase**(U/L)	525.43 ± 148.7	534.42 ± 157.6	494.54 ± 148.9	551.26 ± 131.5	0.08
**Fasting blood glucose **(mg/dL)	82.50 ± 8.6	81.85 ± 8.6	82.52 ± 8.9	83.43 ± 8.2	0.06
**Triglycerides **(mg/dL)	112.93 ± 55.4	96.66 ± 40.1	110.51 ± 52.9	140.16 ± 66.8	<.0001
**Total cholesterol**(mg/dL)	169.60 ± 30.9	161.55 ± 26.7	168.12 ± 31.8	183.45 ± 30.9	<.0001
**HDL-cholesterol **(mg/dL)	45.75 ± 11.8	48.48 ± 13	44.44 ± 10.3	43.38 ± 10.8	<.0001
**LDL-cholesterol **(mg/dL)	101.74 ± 26.6	94.13 ± 23.5	102.12 ± 27	112.53 ± 26.6	<.0001
**Systolic blood pressure **(mmHg)	103.39 ± 14.9	97.78 ± 13.3	104.56 ± 13.5	110.13 ± 15.8	<.0001
**Diastolic blood pressure**(mmHg)	63.85 ± 10.3	61.19 ± 9.1	65.27 ± 10.4	66.01 ± 11	<.0001

*: mean ± standard deviation

**Table 2:** Correlation between waist circumference and cardio-metabolic risk factors with liver enzymes according to sex and body mass index

Variables		Normal weight Group			Overweight Group			Obese Group			Total		
					
		Girls	Boys	All	Girls	Boys	All	Girls	Boys	All	Girls	Boys	All
**Waist** **Circumference **(cm)	**ALT**	0.013	0.142	0.031	0.115	0.027	0.077	0.028	0.230**	0.152**	0.205**	0.408**	0.306**
	**AST**	-0.251**	-0.230**	-0.249**	-0.025	-0.159*	-0.078	0.033	-0.440	0.002	-0.089*	0.065	-0.011
	**ALP**	-0.308**	-0.121	-0.269**	-0.305**	-0.168*	-0.227**	-0.332**	-0.268**	-0.286**	-0.216**	-0.117**	-0.163**

**Systolic blood pressure**(mmHg)	**ALT**	-0.054	-0.118	-0.078	-0.069	0.128	0.048	0.003	0.088	0.072	0.038	0.201**	0.136**
	**AST**	-0.248**	-0.328**	-0.270**	-0.124	-0.054	-0.680	-0.265**	-0.177	-0.176*	-0.197**	-0.83	-0.125**
	**ALP**	-0.075	-0.147	-0.097	-0.224**	-0.258*	-0.181**	0.280	-0.142	-0.044	-0.111*	-0.173**	-0.110**

**Diastolic blood pressure**(mmHg)	**ALT**	-0.018	-0.133	-0.071	-0.199*	0.101	-0.055	0.068	0.127	0.095	-0.031	0.150**	0.060
	**AST**	-0.216**	-0.307**	-0.245**	-0.219**	-0.081	-0.149*	-0.213*	0.025	-0.049	-0.213**	0.028	-0.122**
	**ALP**	-0.122	-0.106	-0.127*	-0.212**	-0.208*	-0.178**	0.127	-0.161	-0.020	-0.112*	-0.160**	-0.124**

**Fasting blood glucose**(mg/dL)	**ALT**	0.002	0.106	0.044	0.179**	0.051	0.137**	-0.048	0.092	0.004	0.068	0.092*	0.086**
	**AST**	0.150*	0.243**	0.181**	0.201**	0.288**	0.244**	0.111	0.159	0.109	0.147**	0.218**	0.181**
	**ALP**	0.051	0.109	0.083	0.200**	0.073	0.171**	-0.093	0.022	-0.058	0.053	0.072	0.070*

**Triglycerides** (mg/dL)	**ALT**	0.109	0.055	0.087	0.172**	0.129	0.149**	0.271**	0.194*	0.218**	0.239**	0.274**	0.249**
	**AST**	0.101	-0.095	0.036	0.138*	0.217**	0.159**	0.136	0.195*	0.160**	0.113**	0.201**	0.148**
	**ALP**	0.122	-0.020	0.061	0.114	0.086	0.095	0.183*	0.091	0.059	0.135**	-0.018	0.077*

**Total Cholesterol**(mg/dL)	**ALT**	0.028	0.030	0.023	0.119	0.072	0.099	0.164*	0.127	0.126*	0.155**	0.210**	0.173**
	**AST**	0.059	-0.013	0.028	0.058	0.049	0.055	-0.015	0.158	0.078	0.091	0.136**	0.078**
	**ALP**	0.193**	0.034	0.113*	0.116	0.058	0.092	0.019	0.088	0.042	0.135**	0.055	0.093**

**LDL-C**(mg/dL)	**ALT**	0.098	0.067	0.081	0.116	0.002	0.068	0.035	-0.021	-0.035	0.143**	0.160**	0.144**
	**AST**	0.051	-0.014	0.023	0.037	-0.085	0.010	-0.115	-0.040	-0.052	0.009	0.043	0.020
	**ALP**	0.136*	-0.012	0.064	0.085	-0.009	0.056	-0.084	0.069	0.029	0.074	0.013	0.044

**HDL-C**(mg/dL)	**ALT**	-0.103	-0.060	-0.088	0.012	-0.108	0.042	0.048	0.040	0.039	-0.067	-0.055	-0.065*
	**AST**	0.006	0.083	0.030	0.032	0.183*	0.083	0.089	0.124	0.110	0.033	0.088	0.052
	**ALP**	0.129*	0.223**	0.157**	-0.025	0.171*	0.020	0.066	0.245**	0.140*	0.074	0.207**	0.111**

*:P value < 0.05; ** P value < 0.01 ALT: Alanine aminotransferase (U/L); AST: Aspartate aminotransferase (U/L); ALP: Alkaline phosphatase (U/L)

**Figure 1:**
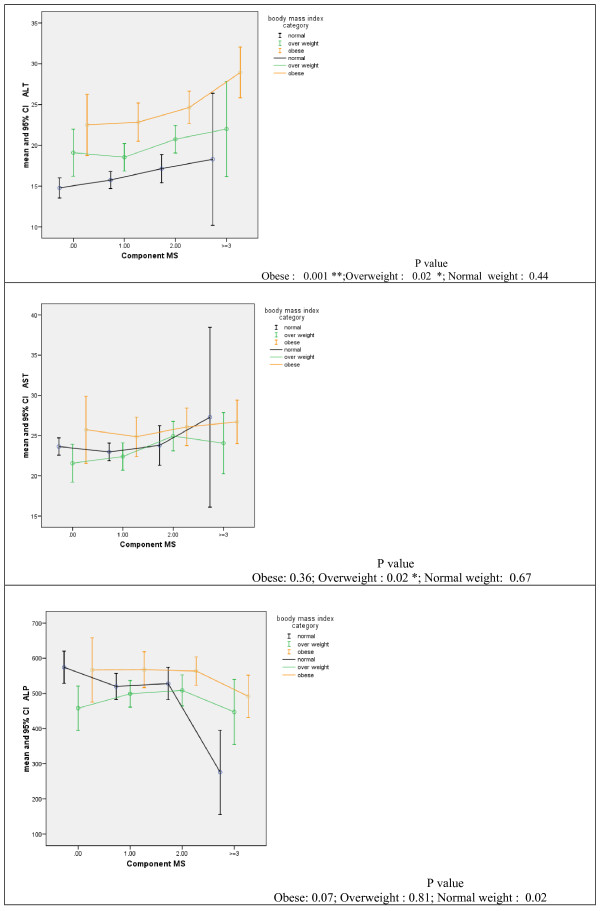
**Mean (95%CI) of liver enzymes according to the number of components of the metabolic syndrome**.

Figure 1 shows the mean values of ALT, AST and ALP by the number of components of the metabolic syndrome in normal-weight, overweight and obese groups. In all three BMI categories, ALT increased significantly by increasing the number of the components of the metabolic syndrome. The corresponding figure for AST was significant only in the overweight group, whereas mean ALP decreased significantly in the normal weight group.

Table 3 shows that in general, odds ratio for elevated liver enzymes and sonographic fatty liver increased significantly with higher number of the components of the metabolic syndrome and higher BMI categories before and after adjustment for age. Among total study population, ORs for elevated ALT or elevated AST in children/adolescents with high FBS, high TG and high total cholesterol were significantly higher than ORs in subjects with none of these metabolic abnormalities. Whereas, associations of low HDL-C and high LDL-C with elevated ALT and AST were not significant.

**Table 3:** Odds ratio for elevated liver enzymes and sonographic fatty liver according to the number of the metabolic syndrome components and categories of body mass index before and after adjustment for age

**Variables**	**Participants n (%) total:1110**		**Unadjusted for age OR (95% CI)**				**Adjust ed for age OR (95% CI)**			
				
		**Participants who underwent sonogrphy n (%) total: 931**	**Elevated ALT**	**Elevated AST**	**Elevated ALP**	**Fatty liver**	**Elevated ALT**	**Elevated AST**	**Elevated ALP**	**Fatty liver**
										
**Number of components of the metabolic syndrome^¶^**										
**0**	210(18.9%)	183(19.7%)	1.0	1.0	1.0	1.0	1.0	1.0	1.0	1.0
**1**	436(39.3%)	377(40.5%)	1.06 (0.05, 2.19)	1.73 (0.81,3.70)	1.34 (0.73,2.46)	2.85* (1.15, 7.06)	1.05 (0.51, 2.17)	1.75 (0.81, 3.74)	1.69 (0.89,3.17)	2.81* (1.13, 6.98)
**2**	383(34.5%)	311(33.4%)	1.61 (0.79, 3.27)	3.18* (1.52,6.67)	1.61 (0.87, 2.98)	2.78* (1.13, 6.85)	1.59 (0.78, 3.2)	3.22* (1.53,6.78)	2.14* (1.12, 4.07)	2.76* (1.12, 6.81)
**≥ 3**	81(7.3%)	60(6.4%)	3.69* (1.61, 8.45)	2.71* (1.05, 7.0)	0.74 (0.25, 2.19)	11.31** (3.9, 32.81)	3.55* (1.50,8.38)	2.86* (1.08, 7.6)	1.49 (0.49,4.59)	9.84** (3.32, 29.15)
**Body mass index categories**										
**Normal weight**	438(39.5%)	389(41.8%)	1.0	1.0	1.0	1.0	1.0	1.0	1.0	1.0
**Overweight**	377(34.0%)	314(33.7%)	2.22* (1.23,4.02)	1.32 (0.79,2.21)	0.87 (0.54,1.41)	10.53** (3.15, 35.23)	2.19* (1.20, 3.96)	1.89 (0.79, 2.25)	1.08 (0.66, 1.78)	10.05** (3.0, 33.62)
**Obese**	295(26.6%)	228(24.5%)	3.41** (1.87, 6.19)	1.92* (1.14, 3.25)	1.11 (0.67, 1.85)	121.84** (37.69, 393.83)	3.41* (1.87,6.20)	1.34* (1.12, 3.21)	1.05 (0.62, 1.76)	127.13** (39.2, 412.26)

^¶^: The ORs (95% CIs) were calculated by performing logistic regression analysis to determine the risk of elevated ALT according to the number of components of the metabolic syndrome after adjustment for age and body mass index.

P value < 0.05; ** P value < 0.01

While in overweight group, ORs for elevated AST or elevated ALT in children/adolescents with high TG were significantly higher than ORs in those with normal TG, the associations between other metabolic variables and elevated ALT, AST and ALP were not significant. In obese group, ORs for elevated ALT or elevated AST in subjects with high TG and high total cholesterol were significantly higher than ORs in those without these metabolic abnormalities Relationship between elevated ALT and the number of the metabolic syndrome components showed that the prevalence of metabolic syndrome components in children/adolescents who had ultrasonographic examinations with 0, 1, 2, and ≥ 3 risk factors were 19.7%, 40.5%, 33.4%, and 6.4%, respectively. The unadjusted ORs for elevated ALT levels in children/adolescents with 1, 2, and ≥ 3 risk factors were 1.06 (95% CI, 0.05-2.19), 1.61 (95% CI, 0.79-3.27), and 3.69 (95% CI, 1.61-8.45), respectively, that was significant for subjects with ≥ 3 risk factors. The unadjusted ORs for elevated AST were significant in children/adolescents with 2 risk factors [3.18 (95% CI, 1.52-6.67)], and for subjects with ≥ 3 risk factors [2.71, 95% CI, 1.05-7.0). Both adjusted and unadjusted ORs for fatty liver were significant in children/adolescents with 1, 2, and ≥ 3 risk factors. While for elevated ALT, the adjusted OR was significant only in subjects with ≥ 3 risk factors [3.69 (95% CI, 1.61-8.45)], for elevated AST it was significant in children/adolescents with either 2 risk factors [3.22 (95% CI, 1.53-6.78)], or ≥ 3 risk factors [2.86 (95% CI, 1.08-7.6)]. The adjusted OR for elevated ALP was significant only in subjects with 2 risk factors [2.14 (95% CI, 1.12-4.07)].

## Discussion

Overweight and obese children/adolescents were much more likely to have elevated ALT, AST and ALP levels than normal weight children/adolescents in our study, a finding compatible with the results of the third National Health and Nutrition Examination Survey (NHANES III) [21]. But the prevalence of elevated ALT in overweight children in the current study was almost double of that in NHANES III. Because of the cross-sectional design of the study, we were not able to define a causal relationship between metabolic syndrome and the levels of liver enzymes. However, our findings agreed with a recent prospective study that revealed AST and ALT could independently predict type 2 diabetes in persons aged 40-69 years after 5.2 years [22]. Our results showed that overweight or abdominal obesity was the most sensitive predictor of elevated ALT levels in children/adolescents.

Still the measurement of the WC has not been included in the routine physical examinations in the clinical and epidemiological studies of the pediatric age group. Several studies have found that WC is a simple and effective indicator that can be used to screen central adiposity [23] as well as cardiovascular risk profiles [24,25] in children/adolescents. Oliveira et al. demonstrated that for each 5-cm increase in WC and every 1-point increase in BMI z-score, there was a 1.3-fold greater chance of having increased ALT levels [11]. Then, WC should consider measuring in adolescents being evaluated for non-viral and nonalcoholic elevation of aminotransferase levels. We found that the metabolic syndrome is an important cause of elevated ALT levels in adolescents. Our study on a subsample of 100 adolescents aged 12-18 years selected from the participants of the current study revealed significant associations between insulin resistance and NAFLD, and similar risk factors and protective factors for these two inter-related disorders [26]. The findings of the current study agreed with other studies that showed NAFLD occurs most commonly in conditions associated with insulin resistance in children [27-29]. Although the ORs did not represent the biological aggravation of liver function, our study revealed that the higher the number of components of the metabolic syndrome, the higher the risk of elevated ALT or AST. Different studies have shown that a number of metabolic syndrome components, obesity and insulin resistance are strong predictors of increased ALT activity in NAFLD in children/adolescents [30-35]. Central obesity, raised TG, reduced HDL, and elevated fasting glucose are metabolic syndrome components that contributed to increased ALT activity. Hyperlipidemia, especially elevated TG concentrations, was a primary risk factor for NAFLD [28] and was also an outcome of NAFLD. We found that high TG concentrations were significantly associated with elevated ALT, AST and ALP in children/adolescents. In future interventional studies, in addition to liver enzymes, high-sensitivity C-reactive protein, gamma-glutamyl transpeptidase, uric acid, vitamin D, adiponectin, ghrelin level, insulin levels, insulin resistance and the oral glucose tolerance test could be the integral part of the evaluation of patients with NAFLD to further clarify the relationships among different biologic factors and metabolic syndrome.

The cross-sectional nature of this study does not allow us to infer causality but rather serves to generate and test hypotheses. The other limitation was that we could not examine the insulin level because of the financial limitations for the large number of study participants. Moreover, IDF has not defined the criteria of the metabolic syndrome in children aged less than 10 years of age, and for the whole participants, we applied the IDF definition for those aged equal or greater than 10 years. We should also acknowledge that we used biochemical and sonographic findings as surrogate markers of fatty liver, none of the participants had clinically significant evidence of hepatosteatosis and we did not confirm fatty liver by histology.

Our findings complement the recent findings about the relationship of fatty liver with the metabolic syndrome in children and adolescents, and emphasize on the role of obesity in this regard. We found that the higher the number of components of the metabolic syndrome, the higher the risk of elevated ALT and sonographic fatty liver. A recent review suggested that ectopic fat accumulation (such as visceral and hepatic fat accumulation) has a pivotal role in the development of the metabolic syndrome [36], our findings on the interrelationship of biochemical and sonographic indexes of NAFLD with the components of the metabolic syndrome and with increase in their number is a confirmatory evidence for such association from early life, it is suggested to determine the clinical impact of such association in future longitudinal studies.

## Competing interests

The authors declare that they have no competing interests.

## Authors' contributions

RK participated in the design, coordination and conduction of the study and drafted the manuscript. SC commented and edited the manuscript draft. AA participated in the coordination and conduction of the study. ZF, SG, AB, HS, NK and PP conducted the study. All authors read and approved the final manuscript.
